# Supplementary material for: Weight Loss Management and Lifestyle Changes during COVID-19 Lockdown: A Matched Italian Cohort Study
Source: Nutrients. 2022 Jul 14;14(14):2897. doi: 10.3390/nu14142897 (PMC9322349; doi:10.3390/nu14142897)
Supplement: Supplementary file 1 [file nutrients-14-02897-s001.zip › supplementary materials.pdf]

Supplementary Materials

Missing Data

Of eligible exposed and not exposed patients, the following variables resulted missing:

- waist circumference, 3.2% missing
- fat mass fraction, 10.8% missing
- weekly metabolic equivalents of tasks, 10.0% missing
- prescribed energy intake, 0.3% missing

While missingness of waist circumference and prescribed energy intake was attributed mainly to data entry errors, non-random mechanisms have partially determined missingness in fat mass fraction and weekly metabolic equivalents of tasks:

- fat mass fraction was missing in patients with skinfold thicknesses > 40 mm for instrument limitations and patients older than the target population of the Durnin predictive formula (68 years for females, 72 years for males):
    - body mass index (kg/m^2) of incomplete cases vs. complete cases: 27.5 (24.0, 32.0) vs. 28.9 (25.7, 33.0), $p<0.001$
    - age (years) of incomplete cases vs. complete cases: 48 (36, 57) vs. 28.9 (25.7, 33.0), $p<0.001$
- weekly metabolic equivalents of tasks were missing in patients older than the target population of the International Physical Activity Questionnaire (69 years), but a clear pattern was not found in relation to age or other variables

A sensitivity analysis related to missingness of fat mass fraction and weekly metabolic equivalents of tasks was warranted and conducted (see below).

As the maximum ratio of missingness was ~10%, 10 imputation datasets were created though multivariate imputations by chained equations, and as all missing variables were numeric variables, predictive mean matching was the only method used. Predictors for the imputation model were:

- demographic variables: age and sex
- anthropometric variables: height and weight only, as other variables resulted redundant

Results from the imputation procedure are shown in Figure A1.

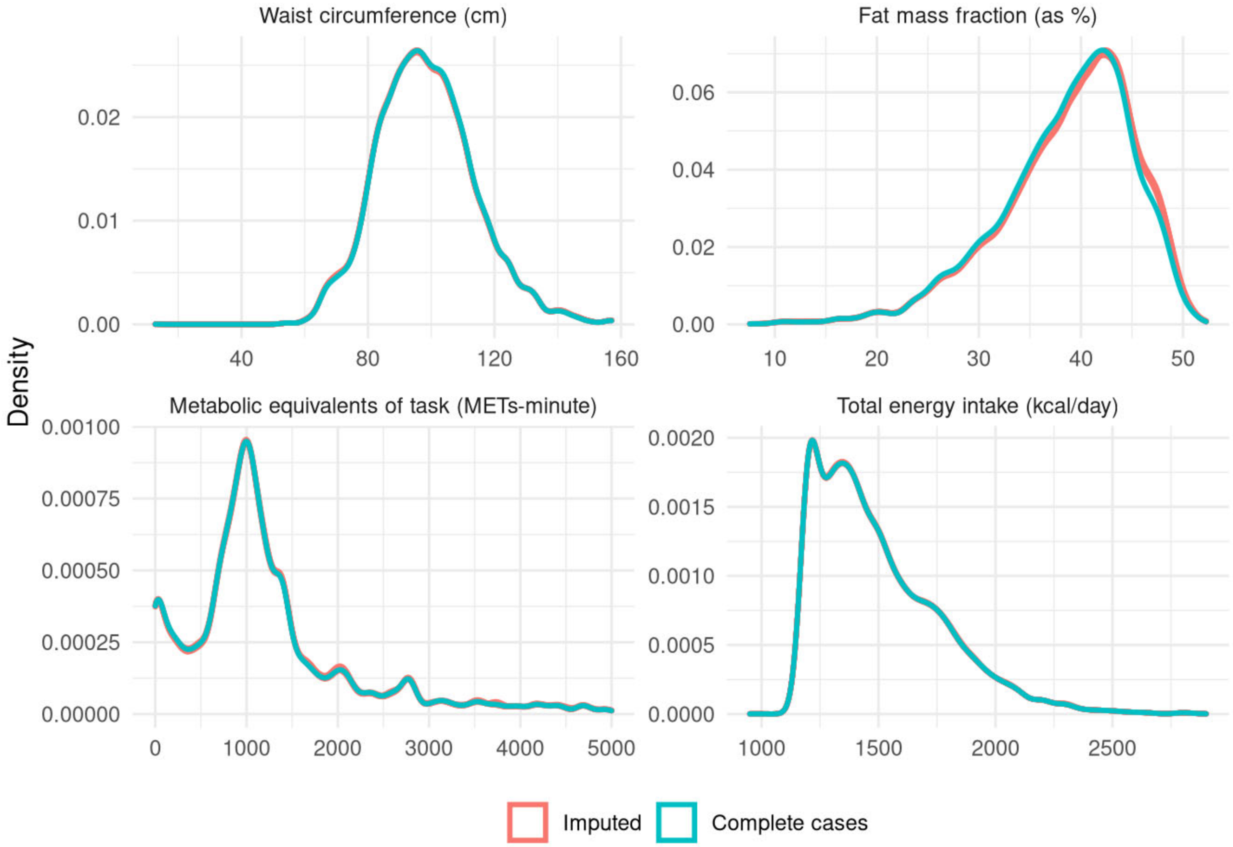

Figure S1: Density plot of observed and imputed data

All variables with missing data could be reasonably be imputed from other related variables included in the dataset
